# Video Intervention Therapy for primary caregivers in a child psychiatry unit: a randomized feasibility trial

**DOI:** 10.1186/s13063-021-05668-w

**Published:** 2021-10-30

**Authors:** Fanny Leyton, Marcia Olhaberry, Javier Morán, Cecilia De la Cerda, María José León, Catalina Sieverson, Ángela Alfaro, Camila Hernández, Rubén Alvardo, Howard Steele

**Affiliations:** 1 Escuela de Psicología, Pontificia Universidad Católica, Av. Vicuña Mackenna 4860, Macul, Santiago, Chile; 2 Departamento de Pediatría, Escuela de Medicina, Facultad de Medicina, Universidad de Valparaíso, Subida Leopoldo Carvallo, 200 Valparaíso, Chile; 3 Programa Salud Mental Perinatal, Red de Salud UC Christus, Santiago, Chile; 4 Escuela de Psicología, Universidad de Valparaíso, Valparaíso, Chile; 5 Departamento de Psicología de la Facultad de Ciencias Sociales de la Universidad de Playa Ancha, Valparaíso, Chile; 6 Milenium Institute for Depression and Personality Research (MIDAP), Santiago, Chile; 7 Program of Mental Health, School of Public Health, Faculty of Medicine, Universidad de Chile, Santiago, Chile; 8 Center for Attachment Research, Psychology Department, New School for Social Research, New York, USA

**Keywords:** Video feedback intervention, Video Intervention Therapy, Parental Reflective Functioning, Inpatient psychiatric children

## Abstract

**Background:**

During child psychiatry hospitalization, working with the families or attachment figures is a challenge, most of the children who are admitted to these units come from multi-problem families, with limited research in this area. Video feedback (VF) interventions have proved to be a powerful resource to promote parental and child well-being in small children and has been used with parents with a psychiatric condition. Parental Reflective Functioning (PRF) is one of the parental abilities that can be improved with VF and could be especially important in coping with conflict and negative emotions in older children. The aim of this study is to implement Video Intervention Therapy (VIT) to enhance PRF in primary caregivers of inpatient psychiatric children. As there is no published research using VF with parents of children with severe psychopathology in a hospitalized context. This report, then, becomes a much needed pilot study providing evidence for a larger randomized control trial (RCT).

**Methods:**

The study is a single-center, two-arm feasibility randomized control trial with a qualitative component. Block randomization was done to generate a 2:1 allocation, leaving more participants in the intervention group. The intervention comprises four modules; every module has both one video-recorded play session and one VIT session (in a group setting) per week. Evaluation of the caregivers included assessments of PRF and well-being, and child assessment included parent-ratings and clinician-ratings of symptomatology and general functioning.

**Results:**

Thirty participants were randomized; eligibility and recruitment rate were 70.6% and 83.3%, respectively. The compliance-to-intervention rate was 85% in the VIT group and 90% in the control group. All participants completed entry evaluation and 90% at the 3-month follow-up. The intervention was acceptable to participants and feasible for therapists to deliver. Outcome data must be treated with caution due to the small numbers involved, yet indicate that the VIT may have a positive effect in improving parental and child mental health outcomes.

**Conclusions:**

VIT for primary caregivers of child inpatient children was feasible to deliver and acceptable for participants, therapist, and the staff unit involved; there is sufficient evidence to undertake a full-scale effectiveness RCT.

**Trial registration:**

ClinicalTrials.govNCT03374904. Registered on 14 December 2017

## Background

Hospitalization in child psychiatry services is the most specialized and expensive step in the pyramid of interventions available to treat severe psychiatric problems in children. Admitting a child should be the last resource not only because of the costs involved, but also because separating the child from his/her usual environment (family, peer group, and community) may be detrimental to the child’s development [1] and the costs and benefits of hospitalization should be analyzed on a case-by-case basis. Nevertheless, there is a growing need for beds and admission rates have increased in the last decades [2–4].

In countries enjoying community mental health care systems, referral of children and young adolescents to hospitalization occurs when there is a lack of response to outpatient treatments. Most of those children have mental disorders compounded by co-morbidities including learning and developmental problems [5]. These children generally live among multi-problem, at-risk families, characterized by parental mental health problems, socioeconomic difficulties, addiction, history of abuse, and neglect, among other problems [6–8].

There is a wide range of family interventions, all of which are effective in managing child mental health issues [9] in an ambulatory context. Any pediatric psychiatric hospitalization unit must necessarily intervene not only with the child, but also with their family. The best way to do this remains an open question. A recent review of evidence-based family interventions in child psychiatry, where the authors summarize 15 years’ work of randomized controlled trials (RCTs), there is no mention of any RCT in a hospital setting [9]. There are different ways to respond to this need in each unit’s “milieu,” including inpatient family units, where one or more members of the family are hospitalized together with the child, where a variety of family interventions [8, 10], and elements of a family-centered care approach are applied [11].

There are few studies about family interventions in an inpatient setting, two of them evaluate the effectiveness of units where the children were admitted with their families, both concluding that the mental health of children and parents improves when using this approach. A limitation of these studies is that because each evaluated the unit in its entirety, a control group was not implemented [8, 10]. A third study implemented universal parent training during the child’s hospitalization and at 3-month follow-up. A positive effect was seen as dysfunctional parenting was reduced and parental mental health improved, but no effect on the children’s symptoms or the quality of interaction between the children and their parents was found [12].

A family or parental intervention during a child psychiatric hospitalization should obviously be effective, but also brief, focused, long-lasting, and tailored to child and family needs and resources. There are three meta-analyses that demonstrate intervention effectiveness, using video feedback (VF) techniques, by improving parental behavior and relationship between parents and children [13–15]. The studies included in these reports mostly include parents of children under 5 years of age, without severe mental health pathologies.

A pilot clinical trial was designed in order to adapt a VF intervention for use during hospitalization and to evaluate future effectiveness. The use of Video Intervention Therapy (VIT) was used since it is a flexible manualized VF intervention, adaptable to different settings and family contexts. VIT aims to enhance parental mentalizing or reflective functioning (PRF) [16–18]. Parental mentalizing refers to a cognitive and emotional process of reflection on the internal mental states of the adult and child in order to regulate the emotional state and act sensitively to the needs of the child [19–21]. Mentalizing has been operationalized in reflective functioning, which can be measured by coding answers to questions that activate the attachment system [22]. It is expected that parents who improve their PRF would not be afraid to reflect on their children’s mental states (thoughts, emotions, desires among others), becoming curious and interested in the different facets of their offspring. They are also expected to develop, through a better internal dialogue, a greater capacity for emotional regulation, which at the same time would allow them to anticipate crisis situations and respond to them in more constructive ways.

### The current study

The objective of this current study is to realize a pilot randomized controlled trial (RCT) to evaluate a Video Intervention Therapy (VIT) to enhance Parental Reflective Function in primary caregivers of inpatient psychiatric children, to assess feasibility and acceptability, and to provide data to estimate parameters required in order to design a definitive RCT as the next step.

## Methods

The protocol describing the methods in detail was previously published [23], and the trial was retrospectively registered on ClinicalTrials.gov NCT: 03374904. This feasibility trial is reported with the Consolidated Standards of Reporting Trials (CONSORT) guideline for pilot trials [24]. Full ethical approval was obtained from the local Ethics Committee (Comité Ético Científico del Servicio de Salud Valparaíso- San Antonio, ORD 1502, date 8-8-17).

### Trial design

The study was conducted from August 2017 to April 2019, being a single-blind, parallel, two-arm, feasibility RCT. Participants were randomized to either an intervention group (4 weekly sessions of dyadic play therapy PT and four weekly sessions of VIT for caregivers) or a control group. Also, a nested qualitative study evaluating perceptions held by caregiver therapist and mental health workers was performed.

### Settings and participants

Participants were recruited from a public child and adolescent psychiatry ward in Valparaíso, Chile, during August 2017 to February 2019. This unit takes care of patients between 6 and 14 years old. There is a previous study describing this unit [25], where it can be noticed that children come from multi-problem families, where an intervention aimed at improving parenting skills could be especially important. Inclusion criteria were (1) being registered as a tutor or primary caregiver during child hospitalization and (2) having a legal or biological kinship with the hospitalized child or adolescent. Exclusion criteria were (1) being foster caregivers or institutional caregivers or (2) having a severe intellectual impairment or (3) having psychotic symptoms or (4) parents who do not provide regular childcare.

### Recruitment procedure

Eligible tutors were invited to participate in the study by a staff member (usually a psychologist, occupational therapist, or psychiatrist). Informed and written consent was obtained from caregivers and assent from children participating in the study, following from this a baseline evaluation was carried out. A record was kept about reasons for refusal to participate or a decision to withdraw. If parents or tutors had further questions, after intake, they were invited to meet with the principal investigator who was the psychiatrist on the ward.

### Sample size

Thirty participants were recruited; the sample size was not chosen to achieve a level of statistical significance, but rather to define key parameters, including feasibility and acceptability, aiming to justify a future and larger RCT [26].

### Randomization and masking

An external researcher performed a blocking randomization procedure using a web-based random number generator, to keep a balance between the number of people who received VIT during the trial extension, 10 blocks of 3 participants were generated. Participants were allocated in a 2:1 fashion, placing the higher number of participants in the intervention group and similar proportions of caregivers in both study arms during the 18-month study period. Only the main investigator was aware of the blocking procedure.

After an entry evaluation was completed in each case, the clinical team was informed by an external researcher as to which allocation that dyad corresponded to, the main investigator informed participants which intervention they were allocated and discussed with them any implication, and it was recorded if participants who were allocated in the control group were disappointed. This was the case for 4 participants.

Three reliable coders, who were masked (outcome assessor masking) and not immersed in the therapeutic context, evaluated PRF level. Caregivers’ identity and allocation group were unknown.

### Interventions

Both interventions were performed by two therapists at a time.

1. Play therapy: all patients and their tutors received weekly dyadic free-play therapy in a 60-min workshop format, coached by a therapist to promote child-oriented and healthy social interactions. A box of toys was available for the children to explore and role play with their caregivers. Also available were rule-based board games and other materials to draw and craft. Games and toys varied according to the child’s preferences and developmental stage [27]. For early adolescents, the therapists encouraged conversations between caregivers and their children, for example negotiation regarding certain topics (e.g., routine at-home visits and eventual discharge).
2. Video Intervention Therapy (VIT) is a six-step video feedback technique that uses behavior-oriented interventions and elements of representational therapy [18]. Videos can be filmed at different settings, with the only requirement being an observable interaction of the child with their caregiver(s) [28].

### Intervention group (play therapy + VIT)

The study includes a four-module intervention; each module contains a play session and a VIT session. During play therapy, a 5- to 10-min film excerpt was made of a child and caregiver interaction. The therapeutic team would then choose approximately 1- to 2-min-long excerpts to display in latter VIT sessions. VIT occurs during the same week of play therapy and VIT excerpts are shown to groups of caregivers, unless there was only one study participant at that time. When VIT excerpts are shown in groups, caregivers view film excerpts of multiple children, not just their own, and actively participate in the session.

The first session of VIT is centrally focused on building rapport with caregivers and reinforcing their observed strengths as seen on the video. The caregiver learns the immediate and longer-term developmental goals for the child from the therapist and other parents. Other caregivers or parents have a unique supportive role to play in a VIT group session because of their peer status. Caregivers, when looking at the videos, may spontaneously talk about something problematic or that they would do differently if they were to find themselves in that situation once again. In other cases, therapists may ask parents if they would want to see something that they may do differently (“negative pattern”). If caregivers agree, they take a deeper look into a negative pattern, using mentalization techniques. The cardinal virtue for the therapist is to assume a non-judgmental stance in working with VIT [18].

A limitation of this model of intervention stems from the dyadic quality of the intervention, such that if a parent needs special unique attention it is difficult to provide this attention as the time when the parent is on the psychiatric unit is typically followed closely, if not cherished, by the child. Unfortunately, in Chile, there is a lack of foster care, with kinship care privileged, so when children must be removed from their families for their protection, they must go to institutions.

### Control group (TAU + play therapy)

Treatment as usual (TAU) includes pharmacological and daycare management, occupational therapy, crisis intervention, psychological counseling, and family assessments and support as needed [6]. Besides that, the participants in the control group received weekly dyadic play therapy sessions as described previously.

### Outcomes

#### Feasibility parameters

1. Eligibility rates: proportion of hospitalization tutors meeting the eligibility criteria
2. Recruitment rates: proportion of participating caregivers, to include reasons for declining to participate
3. Data attrition rate: estimates the proportion of complete entry evaluation by participant and after every session by treatment group
4. Follow-up rate: estimates the proportion of participants completing a 3-month follow-up assessment, per treatment group
5. Treating team’s qualitative assessment regarding intervention, implementation, and delivery

#### Acceptability of intervention

1. Participant-attendance rates: proportion of participants that complete the VIT intervention (4 sessions)
2. Qualitative assessment of the intervention made by caregivers regarding acceptability and satisfaction

#### Secondary outcomes

Demographic and mental health status at baseline and changes over time in PRF, the well-being of caregiver, child symptoms, and general functioning.

### Instruments

A detailed description for the chosen measures is provided in the published protocol.

Caregiver assessment includes:

1. Sociodemographic survey to collect data on the caregiver, child, and family system.
2. Five Minute Speech Sample (FMSS) [29] to assess Parental Reflective Functioning (PRF) with the Reflective Functioning Evaluation Manual [22] that scores PRF in a scale from −1 to 9, 9 being an exceptional PRF. The FMSS was coded by three certificated psychologists with training in Reflective Functioning Scale (RFS) coding. To obtain inter-judge reliability in this sample, all coders coded 20% of the full set of FMSSs. Inter-observer agreement was evaluated with interclass correlation (ICC), obtaining a value of 0.71.
3. General Health Questionnaire-12 (GHQ-12) [30, 31] was used to assess parental well-being with a score from 0 to 36, higher scores indicating a lower level of well-being.

Child assessment includes:

1. Strengths and Difficulties Questionnaire (SDQ) [32, 33] to assess psychiatric symptoms according to caregivers, consisting of 25 items, each uses a 3-point ordinal Likert scale (0 “not true”; 1 “somewhat true”; 2 “certainly true”). Responses are rated 0–2 for negatively worded items and are rated inversely, 2–0, for positively worded items, giving a total score (SDQ-tot) ranging from 0 to 40, where a lower score means less symptoms. In addition, two subscales were used: the one for internalizing symptoms (SDQ-int) regarding emotional and peer items and the one for externalizing symptoms (SDQ-ext) which includes behavioral and hyperactivity items.
2. They were also rated with the Children’s Global Assessment Scale (CGAS) [34, 35] to evaluate general functioning according to therapists, with scores ranging from 1 (most impaired) to 100 (superior functioning).

Qualitative interviews

1. Participants: At the end of the VIT intervention (four completed modules), participants were asked to give an interview, lasting from 30 min to 1 h, conducted by an external researcher. The interview was an open-ended set of questions to explore in-depth caregiver experience regarding intervention acceptance, why they agreed to participate, how they felt as the intervention unfolded, and any workshop difficulties or weaknesses in the format; also explored were changes in the child-caregiver relationship and emergence of new ways of thinking about the child and themselves as parents. The interviews were conducted face-to-face in hospital facilities.
2. Therapists: They answer a 1-h-long interview exploring their experience in intervention delivery, including any time, space, supervision, and collaboration requirements from the inpatient clinical team.
3. Treating team: A nurse, two psychiatrists, and a paramedic were interviewed for 30 to 45 min exploring their experience while the trial was conducted; they were asked to report on their intervention impressions and how the trials impacted the regular inpatient unit functioning. Therapists and the treating team interviews were carried out after the last participant finished the trial.

### Data collection

All participants were assessed at baseline, immediately after every VIT session (or after every play session for the control group), and 3 months after recruitment. There was no compensation given for participating in this trial. Only the SDQ questionnaire was completed at baseline, post-treatment, and a 12-week follow-up.

### Data analysis

#### Statistical analysis

Means and standard deviation, for the whole sample, per main outcomes are presented for both control and experimental groups at baseline, at the end of the intervention, and after the 3-month follow-up. The data sets analyzed consisted of 21 cases, having to exclude all those who dropped out of the study (seven cases), plus a further two excluded due to protocol breaches (one control group participant and one from the intervention group). An analysis of covariance (ANCOVA) was performed to assess between-group mean differences at the end of the intervention and at follow-up, using the baseline as a control variable. Mean change score differences were calculated through *t*-tests for related samples by comparing baseline–post-intervention and baseline–follow-up. In accordance with pilot study recommendations [24], confidence intervals and effect sizes are presented. In the ANCOVA analysis, due to biases regarding the partial *η*^2^ statistics [36], effect sizes were converted to Cohen’s *f*, through G-Power 3.1 [37]. To facilitate result interpretation, contrast analysis mean differences between groups are shown once the baseline scores were controlled. All descriptive and inferential analyses were performed through JASP software, version 0.8.6 [computer software].

#### Qualitative analysis

The grounded theory model was used for this purpose; this is a method of analysis that seeks to describe relevant aspects of a specific field of study, in which theory emerges from the data [38]. The analysis approach used in this study was open code, which corresponds to the inductive process of breaking down data into different units of meaning. This involves transcribing interviews and then analyzing fragments in order to identify key words or phrases (data unit) linking interviewee narrative to the study phenomena. This type of coding implies an emergence of multiple categories, their theoretical saturation, and a deeper data analysis. After constant comparison, analysis, and coding, total saturation is achieved when all data are adapted to emerging categories [39]. Interviews were carried out by the three coders (principal investigator and two child psychiatry fellows) through successive meetings. This had the purpose of triangulating the analyses and reaching an intersubjective agreement regarding categories, concepts, and any properties developed [40]. During this process, an external advisor was consulted regarding emerging results to facilitate findings. The ATLAS.TI v7 software was used for this purpose.

## Results

### Recruitment feasibility

**Fig. 1:**
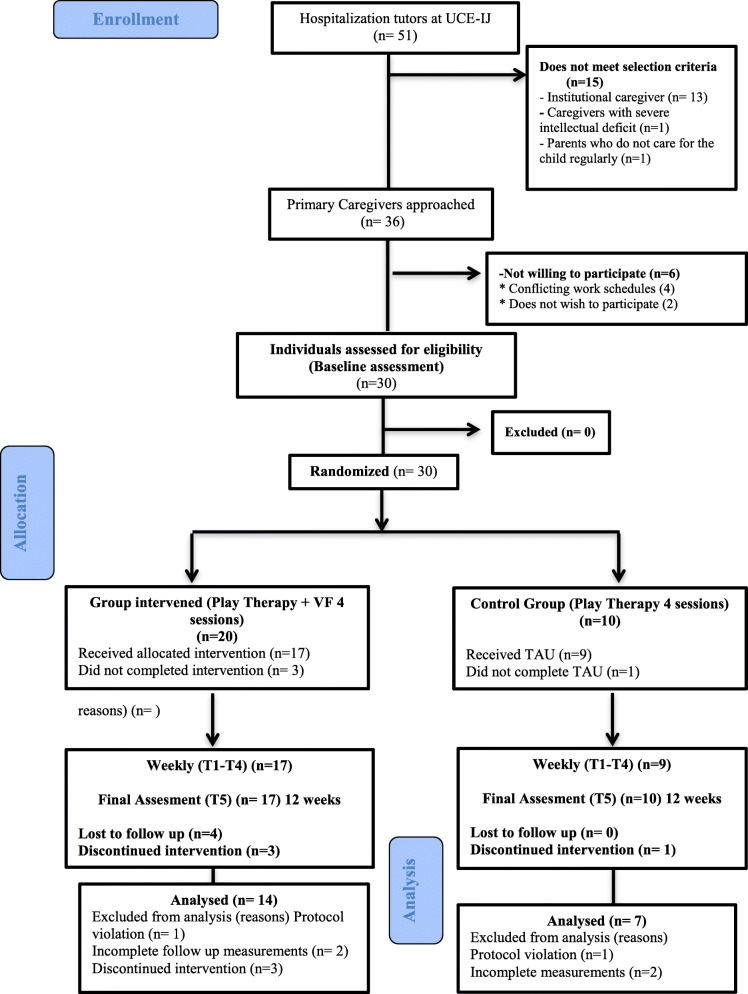
Recruitment and study flow diagram

Participants were enrolled from August 2017 to February 2019; data collection was completed in April 2019. The recruitment and study flow diagram is presented in Fig. 1. During the enrollment period, 51 children were admitted to the inpatient unit, 36 of whom had a primary caregiver who visited regularly during intake, with an eligibility rate of 70.58%. The main exclusion criterion was not having a primary caregiver during their stay, 13 of whom were institutionalized children, with minimal, or no contact with any of their attachment figures. A further two children were in transition to an institution, yet the mother of one of these children refused any kind of family intervention, while the grandparent of the second child suffered severe cognitive impairment. The recruitment rate was 83.33%, 36 eligible cares were invited, and six declined, four of whom were single working mothers with scheduling conflicts and two were not willing to be involved in a research study.

### Delivery feasibility

During the 18-month research period, weekly play therapy and VIT sessions were held, except for a 3-week hiatus. Both workshops were co-facilitated by two therapists, although sometimes only one therapist was present. A total of 4 therapists delivered the intervention, two were available for the initial 10 months of the research period, and the other two were available for the remaining time; all therapists were trained and supervised by certified VIT supervisors. While VIT was designed to be conducted in a group setting, it was common, for at least one session with each caregiver, to occur individually, because there was no other caregiver available at that time. Individual sessions were used to explore caregiver childhood, and to what extent she feels that the way she was raised as a child may influence her experience as a mother.

The compliance-to-intervention rate was 85% in the VIT group (17 out of 20 caregivers) and 90% in the control group (9 out of 10 caregivers). Three caregivers dropped out of the VIT workshop, upset with the inpatient unit team after their decision to file a “child abuse report,” so mothers refused any further intervention. One control group caregiver did not finish play therapy because the daughter refused to see the mother for an extended period of time.

Neither the caregiver nor the child reported adverse effects during play and VIT therapies. No caregiver reported any problems with VIT therapists.

### Feasibility of data collection and outcome measures

All participants completed the entry evaluation, 26 completed the evaluation at the end of the intervention (86.66%), and 27 completed the 12-week follow-up evaluation (90%). Table 1 presents means, standard deviations, and change scores from baseline for the intent-to-treat group. All questionnaires were checked for completion upon delivery, so that staff could encourage participants to complete them in real time and answer any questions they may have. There was a 100% completion rate for baseline assessment, and the overall completion rate was 93.80%. The completion rate for outcome measures for participants finishing the trial (*n* = 27) was 97%.

**Table 1:** Baseline and change data for intent-to-treat

	Intervention group (TAU + PT + VIT)			Control group (TAU + PT)		
	Baseline mean (SD) *n* = 20	Mean change (SD) BL to PT *n* = 17	Mean change (SD) BL to follow-up *n* = 16	Baseline mean (SD) *n* = 10	Mean change (SD) BL to PT *n* = 9	Mean change (SD) BL to follow-up *n* = 9
PRF	2.45 (1.19)	0 (1.62)	0.19 (1.67)	3.6 (1.51)	−0.11 (1.05)	−0.67 (.29)*
GHQ-12	19 (8)	−11.05 (8.14)*	−5.93 (8.59)	22.1 (6.38)	−9.33 (9.42)	−11.11 (6.07)*
SDQ	23.7 (8.68)	−4.88 (5.85)*	−2.25 (5.11)	25.2(8.31)	−4.67 (4.3)*	−6.78 (6.55)
SDQ-ext	12.4 (5.79)	−3.35 (3.2)*	−.88 (3.72)	13.1 (6.42)	−1.56 (3.68)	−9.22 (4.58)*
SDQ-int	11.3 (4.57)	−1.53 (4.89)	−1.38 (3.34)	12.1 (3.7)	−9.89 (3.37)	−3.44 (3.64)*
CGAS	39.85 (15.22)	21.06 (18.32)*^,a^	17.83 (19.42)*^,a^	44.8 (7.58)	16 (11.7)*^,b^	14 (19.44)*

*TAU* treatment as usual, *PT* play therapy, *VIT* Video Intervention Therapy, *PRF* Parental Reflective Function, *GHQ-12* General Health Questionnaire-12, *SDQ* Strengths and Difficulties Questionnaire, *ext* externalizing, *int* internalizing, *CGAS* Children’s Global Assessment Scale, *SD* standard deviation, *PT* post-treatment, *BL* baseline

^a^*n* = 18; ^b^*n* = 10, *p values < .05

### Participant characteristics

Tables 2 and 3 show demographic characteristics and baseline evaluations for the 30 participants included in this trial.

**Table 2:** Caregiver characteristics

	Intervention group (*n* = 20)	Control group (*n* = 10)
Relation to patient		
Mother	15	6
Stepmother	1	0
Grand mother	3	3
Adoptive mother	1	1
Age, mean (range)	43.7 (29–64)	51.2 (32–81)
Years of education, mean (range)	10.4 (4–19)	12.1 (4–17)
Employment status		
Full time/part time	11	3
Unemployed	7	4
Retired	0	1
Homemaker	2	2
Health insurance		
Public exclusive	10	5
Public non-exclusive	10	5
Private	–	–
Marital status		
Married/living with partner	9	6
Divorced	8	1
Single	3	1
Widowed	0	2
Number of siblings, mean (range)	1.90 (0–5)	1.70 (0–6)
Psychiatrist diagnosis*		
None	10	5
Major depression	5	4
Intellectual deficit	1	0
Bipolar disorder	1	0
Personality disorder	3	1
Current psychiatry treatment	4	1

*Caregivers report or from records (clinical formal evaluation was not performed)

**Table 3:** Child characteristics

		Intervention group (*n* = 20)	Control group (*n* = 10)
Sex (female)		10	2
Education			
	Private	3	4
	Public	8	3
	Free exam system	1	0
	Hospital school	2	2
	Special school	6	1
Age, mean		12.3	12.6
	7–10	3	1
	11–13	11	5
	14–16	6	4
Somatic chronic disease		8	0
Cause of admission	Aggression toward others	13	6
	Suicidal ideation	0	1
	Suicide attempt	4	2
	Self-harm	1	1
	Psychomotor agitation	2	0
Days of hospital stay, mean		57.4	75.2
	1 month	3	1
	3 months	15	6
	6 months	2	2
	More than 6 months	0	1
Number of psychotropic drugs, mean		3.10	3.20
	1	1	0
	2	6	3
	3	5	3
	4 or more	8	4
Previous therapy	Individual psychotherapy	6	5
	Family therapy	3	0
	Occupational therapy	4	3
Maltreatment	Physical	5	2
	Neglect	7	7
	Emotional	9	2
	Sexual abuse	5	2
	Bullying	5	3
	More than one experience of maltreatment	9	5

### Change in outcomes

The following are results for primary and secondary outcomes for the 21 analyzed participants (Fig. 1). These results are shown in Table 4

**Table 4:** Primary and secondary outcomes (*n* = 21)

								Change scores and between-group effect sizes^a^							
		Baseline		Post-treatment		Follow-up		Baseline–post-treatment				Baseline–follow-up			
Outcome	Group	*x̄*	*SD*	*x̄*	*SD*	*x̄*	*SD*	*x̄*	*SD*	*MD* [*CI*]	*f*	*x̄*	*SD*	*MD* [*CI*]	*f*
PRF	1	2.71	1.27	2.79	1.05	2.86	0.95	.07	1.77	−2.90 [−1.45, 0.88]	0.12	.14	1.23	.48 [−0.61, 1.57]	0.23
	2	3.57	1.62	3.29	1.38	3.00	2.16	−.29	.76			−.57	.79		
GHQ-12	1	18.71	7.41	8.79	6.38	11.00	6.70	−9.93	6.91	−3.75 [−10.96, 3.46]	0.26	−7.71	7.12	−.34 [−6.65, 5.99]	0.03
	2	23.86	5.27	14.00	8.27	13.86	6.96	−9.86	10.78			−10	6.11		
SDQ	1	21.57	8.76	16.86	8.12	19.50	7.31	−4.71	5.80	−1.04 [−5.94, 3.86]	0.10	−2.07	5.28	3.21 [−1.65, 8.07]	0.33
	2	24.43	10.06	20.00	8.74	18.14	8.34	−4.42	4.50			−6.29	6.78		
SDQ-ext	1	10.86	5.92	7.50	4.33	10.21	4.10	−3.36	3.54	−2.49 [−5.74, 0.76]	0.39	−.64	4.69	1.47 [−1.46, 4.40]	0.25
	2	11.86	7.38	10.71	7.85	9.29	5.35	−1.14	3.98			−2.57	3.89		
SDQ-int	1	10.71	4.48	9.36	4.16	9.29	4.27	−2	3.79	−0.57 [−3.10, 2.86]	0.08	−1.43	3.50	1.49 [−1.67, 4.66]	0.23
	2	12.57	4.31	10.57	2.37	8.86	3.39	−1.35	4.7			−3.71	3.90		

*PRF* Parental Reflective Function, *GHQ-12* General Health Questionnaire-12, *SDQ* Strengths and Difficulties Questionnaire, *ext* externalizing, *int* internalizing, *SD* standard deviation, *Group 1* intervention, *Group 2* control group, *MD* mean difference. ^a^Baseline was used as a covariate to be controlled through ANCOVA analysis

**Table 5:** Clinical team interview description

Profession	Gender	Age	Role at the unit	Experience (years)	Child mental health experience (years)
Psychiatrist	Female	38	VIT therapist and psychiatry	12	10
Psychology	Female	38	VIT therapist and psychology	13	13
Psychology	Female	28	VIT therapist and psychology	3	2
Psychology	Female	26	VIT therapist	3	2
Psychiatrist	Male	63	Chief psychiatry	37	29
Psychiatrist	Male	38	Psychiatry	14	7
Nurse	Female	29	Chief nurse	4	4
Paramedic	Male	28	Paramedic	6	5

#### Primary outcome

The mean PRF score for the VIT group (group 1) increases throughout each measurement, increasing by 0.07 (*SD* 1.77) from baseline to post-treatment and 0.14 (*SD* 1.23) to the 12-week follow-up measurement, while the control group (group 2) shows a decrease in participants’ means throughout each evaluation, falling by 0.29 (*SD* 0.76) from baseline to post-treatment and 0.57 (*SD* 0.79) to the 12-week follow-up measurement. When comparing groups, the mean difference (*MD*) between the change score in groups 1 and 2 at post-treatment evaluation shows that the control group obtained higher *MD* than the intervention group (mean difference = −2.90, [*CI*] = −1.45,.88), with a small between-group effect size (ES) of 0.12. This difference was reversed at follow-up in favor of the intervention group (*MD* = 0.48, [*CI*] = −.61,1.57) with a between-group ES amounting to 0.23.

**Fig. 2:**
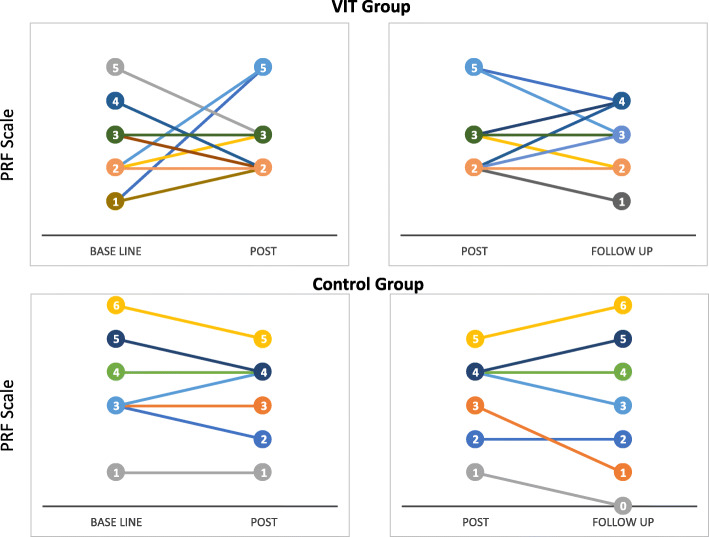
Individual differences in RFS change scores for each group in post-intervention and follow-up

Individual differences in RFS change scores for each group in post-intervention and follow-up can be seen in Fig. 2. There are different individual trajectories in each group; in the control group, a more homogeneous behavior is observed, where variations do not exceed 1 point between one measurement and another, also the majority (6 out of 7) maintained or decreased their PRF after the intervention. A greater variability can be seen in the VIT group both within and between participants.

### Secondary outcomes

#### Caregiver outcome

Parental well-being improved in both groups during the trial. A reduction in the GHQ-12 score of 9.93 is seen in the VIT group at post-intervention, and this difference was smaller at the follow-up (−7.71) when comparing with the baseline. The control group showed similar changes with a mean change score of 9.86 at post-treatment and 10 points at follow-up. Comparison of *MD* between groups, after controlling for baseline, shows that the VIT group had a higher reduction (*MD* = −3.75, *IC* = [−10.96, 3.46]) with a small to medium ES (*f* = 0.26). In the follow-up, again, the VIT group shows a lower *MD* when compared to the control group (*MD* = 0.34, [−6.65, 5.99]) with a small ES (*f* = 0.03).

#### Child outcome

As seen in Table 4, in both groups, all SDQ scales show a decrease in their means at post-treatment evaluation. Mean change score for the VIT group was SDQ-total = −4.71, SDQ-ext = −3.36, SDQ-int = −1.35, while the control group shows similar scores (SDQ-total = −4.42, SDQ-ext = −1.15, SDQ-int= −2). The intervention group showed lower symptomatology scores on comparing means between groups at the post-treatment evaluation, when controlling for baseline. A small effect size is observed in SDQ-total (*MD* = −1.04, *f* = 0.10), a medium-high effect size in SDQ-ext (*MD* = −2.49, *f* = 0.39), and a small effect size in SDQ-int (*MD* = −0.57, *f* = 0.08). In the follow-up, when comparing means for both groups, the intervention group showed lower symptomatology scores than the control group in all SDQ scales, with a medium effect size in both SDQ-total (*MD* = 3.21, *f* = 0.33) and in the SDQ-ext (*MD* = 1.47, *f* = 0.25), while in the SDQ-int, the ES was small-medium (*MD* = 1.49, *f* = 0.23).

### Qualitative outcomes

#### Participant semi-structured interviews

Ten of the 17 participants who completed the intervention agreed to be interviewed. They were six mothers, two stepmothers, and two grandmothers. There was no financial compensation for participating in this interview. Interviews were arranged when the child had an appointment at the hospital, so caregivers who did not continue treatment in the outpatient clinic reported more difficulties in agreeing to the interview.

Two main categories emerge from the analysis: “Workshop Perception” and “Perceived Benefits.” These categories had several subcategories as seen from the caregivers’ narrative. The foundations of these categories are described alongside verbatim quotes illustrating them. Participants are identified in each quote using codes for interview number, relationship, and age.

1. Workshop perceptions: What they thought about their participation

- Motivation to attend: The caregivers report that they accept participation in the trial because they felt it as an opportunity to receive help and because it was a space for dialogue. Some of them also believed the intervention was part of the inpatient treatment.

*“Because I thought: ‘this is the opportunity to help my son’. He had been going to the psychiatrist for a long time, but he did not have a therapist or anything. Then I thought this could be the opportunity; thanks to God it was like that!.”* Interview number 2, mother, 36 years old.

b) Difficulties seen in workshop participation: several caregivers mention various problems in participating, in both interventions (play therapy and VIT). Mentioning aspects such as commuting distances, plus time and money constraints. Two of them failed to understand the workshop purpose. Regarding workshop ambience, they mentioned that during play therapy, if there were any kind of interruptions or when other children were around, the noise became upsetting and it was difficult to focus in playing with their child or also they mention being uncomfortable with being filmed. They also mention that when their child was upset or restless, it became difficult to engage the child in play therapy.

*“Then this other mother was there and I was there with Camilo, then the mother was like, very loud and played with her child, then I did not like that, because I could not concentrate, I was with Camilo and I tried to get into the head of Camilo, but having to work over this mother, I couldn’t do it, but it was the only time I got complicated.”* Interview no. 1, mother, 30 years old.

iii) Positive workshop references: All those interviewed mentioned positive aspects of the workshop: they liked the number of sessions, the group setting, and the video feedback. The latter because they had the opportunity to see themselves interacting with their child, to observe behaviors that are not normally noticed in daily life, particularly regarding positive aspects on mother and child behavior. Video observation also allowed them to become aware of any improvements seen during the process.
iv) Concerning therapists, cares said that they felt listened to and that they were warm and flexible, providing sound advice.

*“Obviously we don’t see oneself, I mean, one doesn’t see the way one acts and cannot know that one is behaving incorrectly. But if one sees oneself in the film, then we do realise it, and it is amazing! One goes through life, strong and straight… and without the video saying, “look, this is you” one cannot reflect, one cannot realise that one is doing something badly. The film made me realise that something was wrong.”* Interview no. 3, stepmother, 58 years old.

e) Suggestions for change: Some of them said they would prefer more VIT sessions, include it in the outpatient setting and incorporating the child and/or other family members, such as either the father or mother, or both (many caregivers were grandmothers). Regarding play therapy, carers prefer interacting with their child in a quiet and private space and involving more child unit staff members. In relation to the trial, they request greater depth when explaining the rationale behind the instruments, specifically FMSS, because they found it difficult to understand why it was necessary to answer the same questions every week.

## Perceived benefits

- In general, carers reported that VIT was an effective help, which supported them during their child’s hospitalization. Support came both from therapists as well as from other parents during group sessions, as they felt understood by them. These participants helped them to see aspects in the videos that they could not see by themselves, including positive interactions between them and the child, and this proved to be, in a crisis setting, both comforting and encouraging.

*“They help me to find the right words to say in certain situations, I don’t know…, for example how to react to Esteban’s anger, because when he is angry the situation is not exactly rosy. When he was enraged, he could kick anybody passing in front of him, he could hit the wall with his fists. In this, they help me a lot, how to contain him, how to handle him”*

Interview 7, mother, 29 years old.

b. Identify parenting problems: Caregivers mention that, through looking at their interactions with their child in the video, they identified certain situations at home where they could act or feel different about their children. All situations mentioned by them were organized in two main themes, difficulties in expressing emotions and difficulties in setting rules and boundaries.
  i. Emotional expression: They reported that sometimes they could overreact vis-à-vis the child’s behavior and get angry very easily, not paying attention to the child’s feelings, not expressing love to the child, describing the child in light of their negative aspects, and not sharing with them any play spaces in the home.
  *“In the past, I couldn’t control myself… For example, I used to shout, I could yell at him and on occasions I could hit him. Then I would turn around, my husband would go to calm Juan and I would go to cry in my room, full of remorse… because I know I should not hit him. But not now, …and I say to myself, “My Goodness, I have changed!”.”* I2, mother, 36 years old.
  ii. Setting rules and boundaries: Being authoritarian, or to give them anything they want, not being clear in rules (or contradicting the norms), to allow the extended family taking decisions that should be taken by her, not intervening when another adult in the home ill treats the child and failing to set boundaries due to fears of how the child might react.
  *“Because, for example, if he wanted an ice cream, I would give him the ice cream. If he wanted me to stand on my head, I would stand on my head, if he wanted to go goodness knows where, I would take him there. The whole family behaved like that, his daddy, the older sisters; whatever he fancied, it was given to him, to avoid him throwing a tantrum, to avoid him breaking things”* I5, mother, 50 years old.

iii. New strategies: Together with becoming aware of their parenting difficulties, they start to practice new driving behaviors

i. Communication: Acknowledge the problem and talk about it, without disqualifications. Providing positive reward, taking into account the child’s opinions, identifying any preferences, finding out common interests, giving explanations about what the child does not understand, and sharing with him what the adult thinks and feels.

*“Now I sit with her, or we go for a walk. ‘D, what is the matter? I feel there is something wrong, I know you’ … ‘I don’t think it is nothing …” “I feel something is happening, …tell me, perhaps I could help you, let’s talk”* I3, stepmother, 58 years old.

ii. Related to affections and emotions: Keeping calm before reacting. Imagine what the child might be feeling, finding ways to calm the child (first, by calming herself, then distracting them and connecting with their emotions), avoiding escalating aggressions, pondering and repairing negative reactions against the child.

*“I say to him, ‘. Jorge, I am fed up with you. Why don’t you go to hospital, I am tired with you, obey me…’ and then, I reminded myself! And I thought ‘No, something might be happening to him’. So, I said to him, ‘forgive me that I shouted at you, sit down, and tell me, what is the matter?’ He said, Mum, I wanted to do this or that, and I said, ‘We’ll do it later, on our return; I have to go out and I cannot leave you alone, we will go out, we will come back and then you will do it’. He replied, ‘Okay’. I realized then that, before, he would have shouted, ‘I’ll do nothing at all!’ and he would have been distressed.”* I2, mother, 36 years old.

iii. Related to rules and boundaries: Defining the rules and respecting them, being flexible vis-à-vis child’s needs, setting boundaries through dialogue, keeping the child apart from conflictive situations, avoiding children’s involvement in adults’ problems, and intervening in case of verbal aggression to the child by other adults.

*“I am not any longer the way I used to be. I was permissive, I used to accept anything, I kept quiet - Not any longer; I say whatever I need to say, I do whatever I need to do.”* I8, grandmother, 59 years old.

- Changes in the way the child is perceived: They feel that they understand better the child’s problem, they are able to acknowledge positive changes, and they can put themselves in the child’s place when the child is feeling poorly, when the child is insistent in certain demands or behaves inadequately. They manage to read better the child’s body language, thus being able to identify better their different emotional states.
  *“It happened that I did not know how to contain her, I didn’t realize that she did all that so as to be taken into account … now it’s better because I have a psychologist, the lady doctors, the play time. Thus, I spend more time with her, I ask her questions about her, …if she feels good or poorly…”* I9, stepmother, 61 years old.
- Family repercussions: Carers reported that they could transmit to the rest of the family what they had learned in the workshop, and they could observe the positive changes occurring in the family, such as showing more respect for each other, expressing better their loving feelings, and finding more spaces to share. Some mothers reported that they recovered their authority within the family.
  *“My husband also learned how to control himself, because before he would shout in anger. Now with just one look, ‘go talk to him’, goes upstairs and then he comes down already calmed”* I2, mom, 36 years old.
- Persistence of the changes: Carers gave concrete examples about their ability to transfer what they had learned in the workshop, while the child was hospitalized, to their daily life at home after being discharged.

*“Last night for example, during Nina’s homework time, I realized that something was wrong with her […] because of her facial expression, her gaze… I understood and then I talked with her.”* I8, grandmother, 59 years old.

### Inpatient unit team interviews

These interviews contribute to a better understanding of delivery feasibility (therapist interviews) and implementation feasibility at an inpatient child psychiatry unit (team unit interviews), and the persons interviewed are described in Table 5.

#### Therapists’ interviews

Four therapists were interviewed, two of them were classified as senior, because they had greater clinical experience and were qualified VIT teachers. The other two were junior therapists as they were under training and with supervised VIT by senior therapists. The open code used in these interviews allowed for the emergence of these two categories.

1. Motivation and perceived benefits: For therapists, participating in these interventions gave them the opportunity to learn a new technique; by learning about VIT, they realized it was a useful tool for going beyond diagnosis and to have a more sensitive understanding of the child, and build a better knowledge of the carer’s upbringing and the way their life history may have affected their relationship with their son. There was also a favorable impact at work with the extended team as every week the processes of each dyad were reported in the team meeting. Besides, paramedics also participated in the games workshop. The two junior therapists valued the opportunity of gaining clinical experience and senior ones felt challenged for bringing into practice their creativity and flexibility.

*“Compared with what I have done before, this was a completely different experience. In general, one approaches parents with certain ideas about how they should change or deal with children. There are only a few opportunities to work together with parents, examining our own thoughts and, progressively, understand parents’ anxieties, expectations… which they might transfer to their children. At the same time, understanding what obstacles there might be to visualize the child’s need. Previously, with my university background, I used to think, ‘blimey! why this dad is not doing this or that’. And one also understands the child’s vulnerabilities from the point of view of certain interactions and not necessarily as something that is intrinsic to them.”* Young therapist.

2. Conditions necessary to practice VIT: The therapists reported:
3. (a) The need to program the necessary time (extra time is required to analyze videos and prepare the workshop; they concluded that this required 30 to 60 min of work per week, according to the number of carers). (b) Technical supervision, which can be scheduled for according to the professionals’ prior training and knowledge about VIT (young therapists also had the requirement of accepting weekly supervised sessions, during which the videos were analyzed by the whole team). This resulted in an extra hour per week to prepare the workshop.
4. (c) Technical requirements such as Internet access, cameras or cell phones, and play materials.
5. (d) Collaboration from team members in preparing the workshop room, to avoid interruptions to the play process (with tasks such as blood pressure checking, administering medicines, among others) and, most importantly, be available in case the primary caregiver is not present for the play workshop or any other contingency (for example in case of child agitation or aggression between peers).

*“These were some situations that occurred during the workshop. The children were playing, as part of the therapy process, and suddenly it was mealtime and the assistant workers would arrive with the food. From the point of view of the workers, ancillary, nursing or paramedic staff, this was never an interruption, but from the therapeutic point of view, there was a clear interruption of a process.”* Senior therapist

#### Treating team

The ward nurse, the play workshop paramedic, and two psychiatrists were interviewed. Reponses were classified into two groups, according to the generated codes: (1) repercussions regarding the running of the ward and (2) difficulties and suggestions regarding implementation.

1. Regarding the ward running: the group described some benefits of VIT including adding to inpatient services, a new psychotherapeutic intervention, giving paramedics an opportunity to participate with the children in a therapeutic activity, delivering relevant clinical information, and, finally, being part of valuable team work. Regarding the research itself, both psychiatrists considered the above as contributing to the operation and dissemination of the unit’s work. There were concerns concerning the control group, but the fact that everybody participated in the play workshop meant that all children had the same daily routine. Some parents requested attending the VIT workshop, which was allowed once the study had finished.

*“In my opinion, the greater change occurred in parents; not so much in the unit - the impact was stronger for parents; they were a bit more aware… daddies appeared to be more involved during the child’s hospitalization, it seemed that their commitment with their children was different”* Paramedic

2) Regarding implementation difficulties, they mentioned that prior to the study start there were problems in the working atmosphere, not everybody in the team knew or fully understood study objectives; but they gradually did so as the research progressed and they involved themselves more and more after witnessing the changes seen in children and their families. They reported that not all the nurses or paramedic showed the same degree of commitment, some collaborated more than others. They are of the opinion that this intervention would be more successful in outpatients, as they are more stable, clinically.

*“A brief training is necessary [in all cases], as the staff is not always the same, there are temporary staff – they may try to do the utmost, but sometimes they don’t have the knowledge or the understanding about what to do or not to do. We have had cases where the mother is playing with the child, and then the paramedic instead of standing by, he/she intervenes in the play, and of course, this alters the dynamics”* Chief nurse.

## Discussion

The present study sought to evaluate the feasibility and acceptability of VIT intervention to enhance PRF in primary caregivers of children in an inpatient psychiatric unit. Quantitative and qualitative methods were used to determine the plausibility of a progression to a full-scale multicenter RCT.

Eligibility and recruitment rate were 70.6% and 83.3%, respectively. This is similar or better to other RCTs using VF to improve parenting [41, 42]. The former compares favorably with other trials delivered in child inpatient units, for example, it fared better than the Rimehaug et al. study of 2019 [8], where recruitment was 67.5% and eligibility was not reported, yet fared worse than Ise et al. trial (2015) [10] where a 91% eligibility and 93% recruitment were obtained. A third of the children admitted to the unit were institutionalized, which became the main reason for not being eligible. Refusal to participate in the study was attributed mainly to difficulties in attending due to conflicting work schedules. Only a few working parents had paid work leave. Hence, we introduced flexibility as the study progressed, scheduling VIT sessions to facilitate participation and adherence.

The instrument completion rate for the set of instruments was high, with minimum instrument loss, possibly because they were applied and checked for completion at each and every assessment point. In the qualitative interviews, it was observed that some caregivers had difficulties in understanding the relevance of answering the same questionnaires and the FMSS at the end of each session, which could affect the quality and richness of their responses.

There were no issues seen in randomization although four caregivers in the control group said they would have preferred to be in the VIT group, and they were offered to participate in the video groups after completing the study. One case was excluded from the analysis due to having received a PRF-based intervention which analyzed what was observed during play therapy.

The completion rate was high in both groups, although lower in the intervention group (85% v/s 90%). The only reason for dropping out from VIT was when the professional had to file a “child abuse report,” in this specific case, resulting in a loss of custody of the child for physical abuse and severe neglect. As can be seen in the results, the frequency of abuse and neglect is high in hospitalized children, so it would be interesting in the future to identify those caregivers at risk of “abuse or neglect.” In these latter cases, it is possible that a protocol modification is required, namely, more sessions, with individual sessions at the beginning aimed at working on motivation and rapport. In those cases where a “child abuse report” should be filed, psychological support or treatment for the adult ought to be included, since even if the child is referred to an institution, he or she will continue to be in contact with their attachment figures. There are some attachment-based interventions for multi-risk families that have achieved successful outcomes with ten sessions [43] and even with six sessions [44] which included home visit sessions. Completion rates are improved when individual sessions are in flexible time or at home visits [16], but home visits were not part of this trial.

The aim of our VIT program is to be part of a larger treatment, each dyad having different therapeutic goals in terms of needs, resources, and phase of their ambulatory treatment. Caregivers with disorganized attachment or with a history of trauma will presumably need a longer intervention.

### Study acceptability

Qualitative interviews showed that caregivers value the empathic and sensitive attitude seen in therapists and the group support achieved. Caregivers also differentiate what they achieved from VIT vis a vis other interventions. Participants and therapists valued the group sessions for their emotional containment of the other participants, and reflections from other caregivers regarding what is seen on the videos made it easier for them to relate, understand, and accept.

The whole group looked for strengths of each other and themselves during the interaction seen in the videos with a non-judgmental attitude, something actively sought by therapists. The shared stories seemed to become a community value, having a unique worth for families who have experienced trauma [45]. Other VF interventions also highlight the group processes as a key component of program’s impact [46]. The positive evaluation of video use was clear in several instances, seeing themselves and how they evolve session after session. Moreover, the power of video is shown by seeing children in a different light, glossing over symptoms and behavior problems to observe constructive behaviors, often unexpected in them, such as collaboration, positive emotionality, and respect. The subjective experience of interviewees referred to different benefits of VIT, such as reflecting on parenting problems (no longer focusing on the children’s problems), developing new behavioral management and emotional regulation strategies that they put into practice when the child was at home. Overall, it was possible to access the subjective experience of the caregivers who participated in this intervention. They saw this space as an opportunity for obtaining knowledge about themselves and their children, valuing understanding and acceptance, acquiring new skills for parenting, and feeling able to put them into practice. The mere fact that some mothers are able to feel “more capable” justifies these brief and tailored interventions. The work of Berthelot et al. [47, 48] shows a strong concordance between mother and child insecure and disorganized attachments, indicating an intergenerational transmission of attachment in parents with childhood histories of abuse and neglect. They emphasize the importance of trauma-specific mentalization, suggesting that it is not the experience of trauma, but the absence of mentalizing regarding trauma that underlies this transmission [48].

Interviews of the treatment team highlight the relevance of having support from the head psychiatrist when conducting the trial, because there were apprehensions before the trial start, such as changes in unit function and excess workload. Toward the end of the study, the nurse, paramedic, and staff psychiatrist valued the intervention in its contribution to their clinical work, and in the acquisition of deeper knowledge and understanding about dyadic dynamic, while parents were more involved in their child hospitalization. Although the study could have generated a greater workload for the staff members, this was not pointed out by them at the end. For future studies, it will be important to improve the induction process for staff and inform the objectives, the theoretical foundations, and the development of the intervention. It is expected that this will generate greater involvement from the rest of the team members and effectively incorporate newcomers. This then becomes an opportunity to reinforce training in child mental health for nursing staff.

From the interviews with the therapists, it appears that supervision is always required, but the frequency varies according to the therapist’s experience. Young therapists were required to have weekly supervision in order to discuss and prepare the workshop; this needs to be considered in a larger study, including theoretical training in the technique plus 1 or 2 h of group supervision per week.

### Change in outcomes

Basal levels in PRF were low; lower than 4 means a failure in mentalizing their children; that pattern is common in persons with borderline personality disorders [49], but also in other disorders such as depression, eating disorders, and obsessive-compulsive disorders among others [50].

When comparing groups at the end of the intervention, the control group shows higher levels of PRF than the intervention group, but at the follow-up, that difference was reversed, both with a small ES. It was expected, to a certain extent, that by the end of the intervention caregivers would have shown a decrease in PRF, since group VIT was a source of emotional support for them, as expressed in qualitative interviews.

Several caregivers mention the desire for a longer intervention that continued on an outpatient basis. Follow-up was made 12 weeks after recruitment, most of the children were either discharged by then or at least had some home leave permits. It is anticipated when children return home that parental stress will increase, generating temporary decreases in parental and child emotional regulation until they have re-adapted to functioning at home. It seems, therefore, that the effect of VIT continues after returning home. The parents in the VIT group became aware of different problems linked to upbringing, as can be seen in the qualitative interviews, so it is possible to suggest that it is at discharge when they display what they have learned during the intervention. PRF is a meta-cognitive skill susceptible to modification with therapy; it has been demonstrated that people with lower levels will need prolonged therapy to modify their functioning [51]. Although the Reflective Functioning Scale (RFS) ranges from −1 to 9, it is an ordinal scale; in clinical terms, a difference of one point accounts for different clinical situations, especially if one moves from non-mentalizing states (score 3 or less) to mentalizing states (score 4 or more) [22, 52].

Regarding secondary outcomes, the results in SDQ scores deserve special mention. In both groups, there is a decrease in SDQ scores and improvement in functioning according to C-GAS. Similar to what was observed with PRF, when comparing groups at the end of the intervention, the control group shows a greater decrease in the three scales, with small ES for SDQ-total and internalizing, and medium-high for the externalizing scale, but at the follow-up, those results were inversed in the three scales, with medium ES. These results are clinically relevant, since the intervention is focused on the parents, so a potential effect at the child level is promising. It will be useful to evaluate the mediation of PRF in these changes, since SDQ was answered by parents. It is therefore possible, on the one hand, that VIT parents were able to reflect in greater depth on their children’s behaviors and emotions, evaluating as less disruptive any externalizing behavior, as well being more aware of unseen internalized problems. On the other hand, it is possible for caregivers to respond more sensitively to the child’s needs, with a better capacity for co-regulation between them. In this way, an enriched bond between caregivers and children can allow this relationship to function as the necessary scaffolding for children to improve their symptoms. Based on this, for future studies, it will be important to include tools that evaluate the children’s perspective and attachment.

### Strengths and limitations

The study combines quantitative and qualitative methodologies, which are recommended for feasibility studies and for research involving psychotherapeutic interventions. Caregiver and child variables were included, such as PRF measurement, a highly relevant parental competence that has been underreported in studies using video feedback [13]. Several actors were interviewed in depth, which allowed us to evaluate acceptability and feasibility from different perspectives. The study showed the utility and clinical value of VIT as a tool for caregivers’ intervention in children having severe psychiatric disorders. The reduced number of sessions allows for adaptation to restricted public health system resources. Therapists rapidly learned VIT techniques through supervision and training.

One shortcoming of the study was the omission of qualitative interviews with the caregivers who participated in the control group. Another limitation is that several parents reported that they would have appreciated being part of the VIT group. Perhaps in a larger study, the control group should participate in another type of parent meeting. The change in the main outcome was less than expected; there are different possibilities that can be explored in a future RCT to better address change in FRP, and one option is performing a longer intervention, since parents with psychopathology, history of trauma, and low baseline levels of pre-mentalizing FRP may require a longer intervention lasting a minimum of 6 sessions. Another option is to change the way FRP is measured, making it better at measuring short-term changes. The chosen instrument, the FMSS, is a brief instrument that parents answer alone in front of a tape recorder, but it is likely that in these parents, changes in the FRP are better observed in interviews or by measuring mentalization in interaction with the child, for example through the Reflective Parenting Assessment, implicit when interacting with school-aged children using an adaptation of the Squiggle paradigm developed by Winnicott in 1968 [53] or using the gold standard measure of reflective functioning, i.e., the Adult Attachment Interview or the Parent Development Interview.

Repeated measurements of the main outcome were made in order to do a larger study with a multilevel design, but there were no advantages in performing repetitive measurements on PRF, so there is no need for these measurements in full-scale trials. A future study could include a direct assessment of recorded play therapy, which can be done in each session, to evaluate the behavior and emotional regulation of caregiver and child, as well as the quality of the dyadic interaction. Finally, in a future study, it will be important to perform a formal assessment of mental health problems in caregivers and early trauma experiences [54].

One of the limitations of the study was not including children in state custody. That could be addressed in a future study by including four sessions of work with a significant figure in the residential center. During the trials, clinical work using VIT was conducted with educational tutors in the child’s residence showing positive clinical results.

## Conclusion

In summary, the current study presents the first evaluation of VIT for caregivers in an inpatient child psychiatry unit. The clinical trials were proved feasible to conduct and indicate that it is possible to undertake a future multicenter study to evaluate the effectiveness of VIT on child and caregiver health outcomes, but it will require some modification of the study protocol according to the feedback generated at the feasibility stage.

## Data Availability

The datasets used during the current study will be available from the corresponding author on a reasonable request.

## Abbreviations

PRF: Reflective Parental Functioning
VF: Video feedback
VIT: Video Intervention Therapy
RCT: Randomized clinical trial
FMSS: Five Minute Speech Sample
GHQ: General Health Questionnaire
CGAS: Children Global Assessment Scale
SDQ: Strengths and Difficulties Questionnaire
